# Supplementary material for: Tracing the Lineage of Two Traits Associated with the Coat Protein of the Tombusviridae: Silencing Suppression and HR Elicitation in Nicotiana Species
Source: Viruses. 2019 Jun 28;11(7):588. doi: 10.3390/v11070588 (PMC6669612; doi:10.3390/v11070588)
Supplement: Supplementary file 1 [file viruses-11-00588-s001.zip › viruses-498781-supplementary.pptx]

## Slide 1
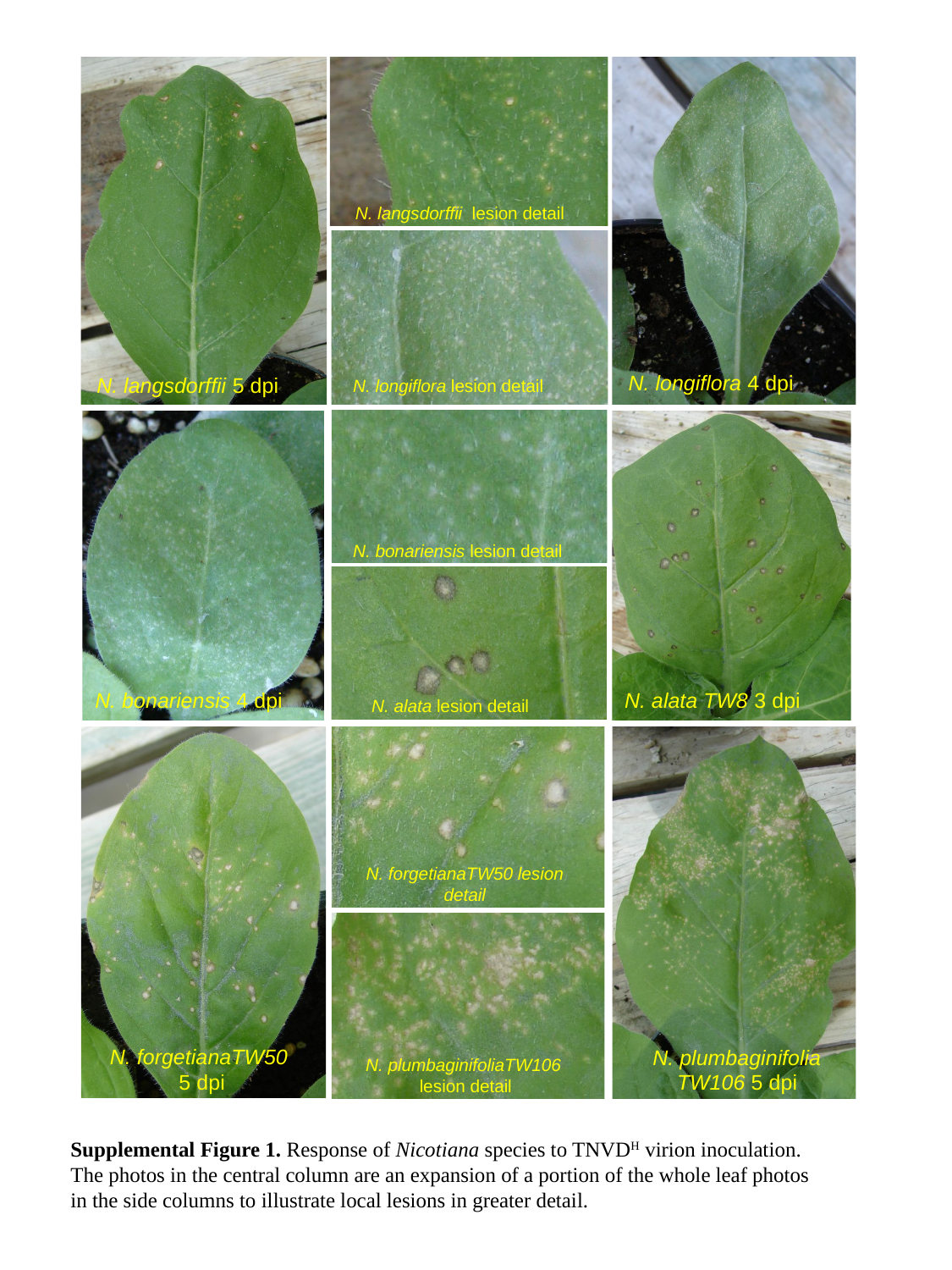

N. langsdorffii lesion detail
N. longiflora 4 dpi
N. langsdorffii 5 dpi
N. longiflora lesion detail
N. bonariensis lesion detail
N. alata TW8 3 dpi
N. bonariensis 4 dpi
N. alata lesion detail
N. forgetianaTW50 lesion detail
N. forgetianaTW50
5 dpi
N. plumbaginifolia TW106 5 dpi
N. plumbaginifoliaTW106 lesion detail
Supplemental Figure 1. Response of Nicotiana species to TNVDH virion inoculation. The photos in the central column are an expansion of a portion of the whole leaf photos in the side columns to illustrate local lesions in greater detail.

## Slide 2
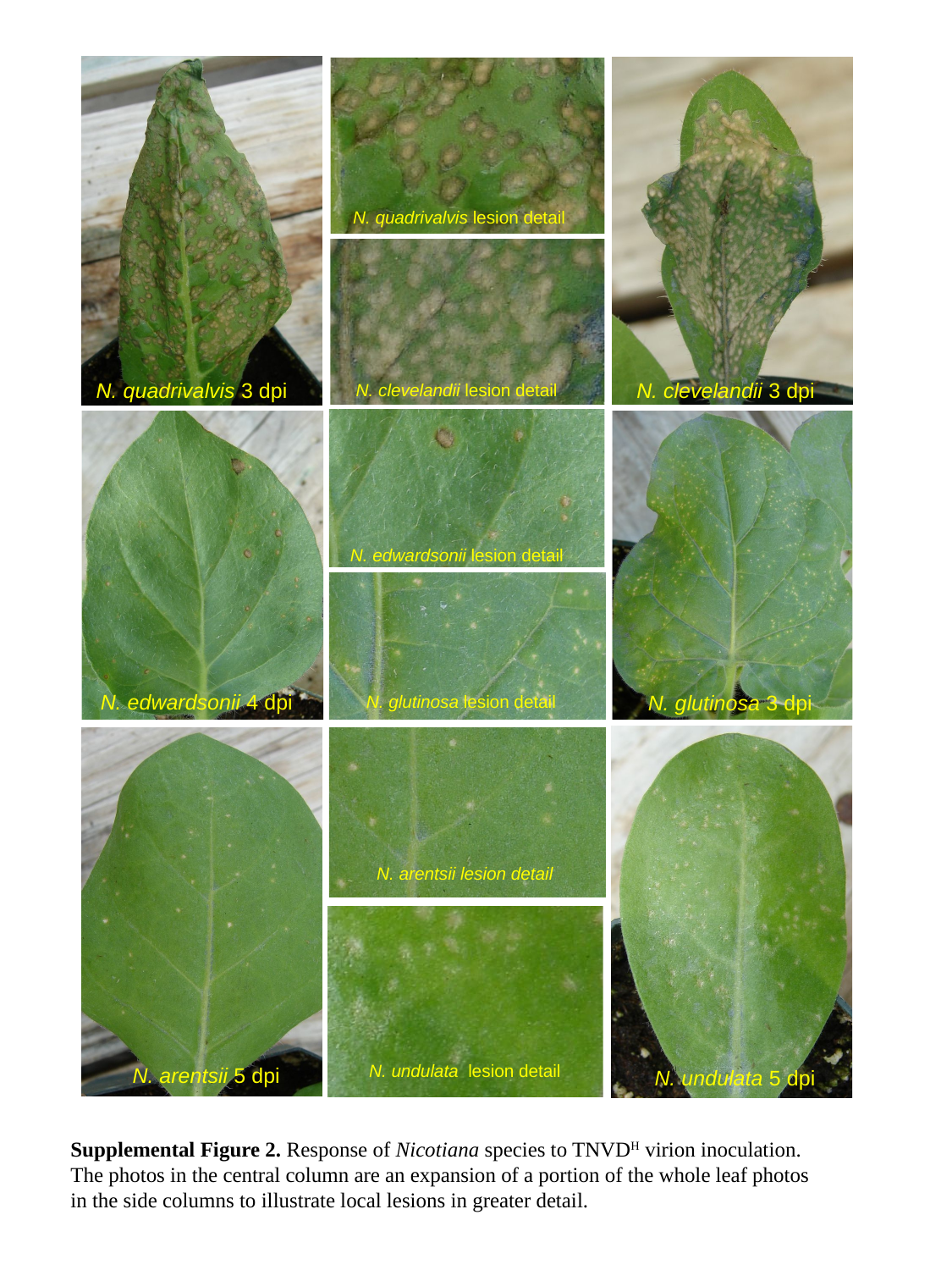

N. quadrivalvis lesion detail
N. clevelandii 3 dpi
N. quadrivalvis 3 dpi
N. clevelandii lesion detail
N. edwardsonii lesion detail
N. edwardsonii 4 dpi
N. glutinosa 3 dpi
N. glutinosa lesion detail
N. arentsii lesion detail
N. undulata lesion detail
N. arentsii 5 dpi
N. undulata 5 dpi
Supplemental Figure 2. Response of Nicotiana species to TNVDH virion inoculation. The photos in the central column are an expansion of a portion of the whole leaf photos in the side columns to illustrate local lesions in greater detail.

## Slide 3
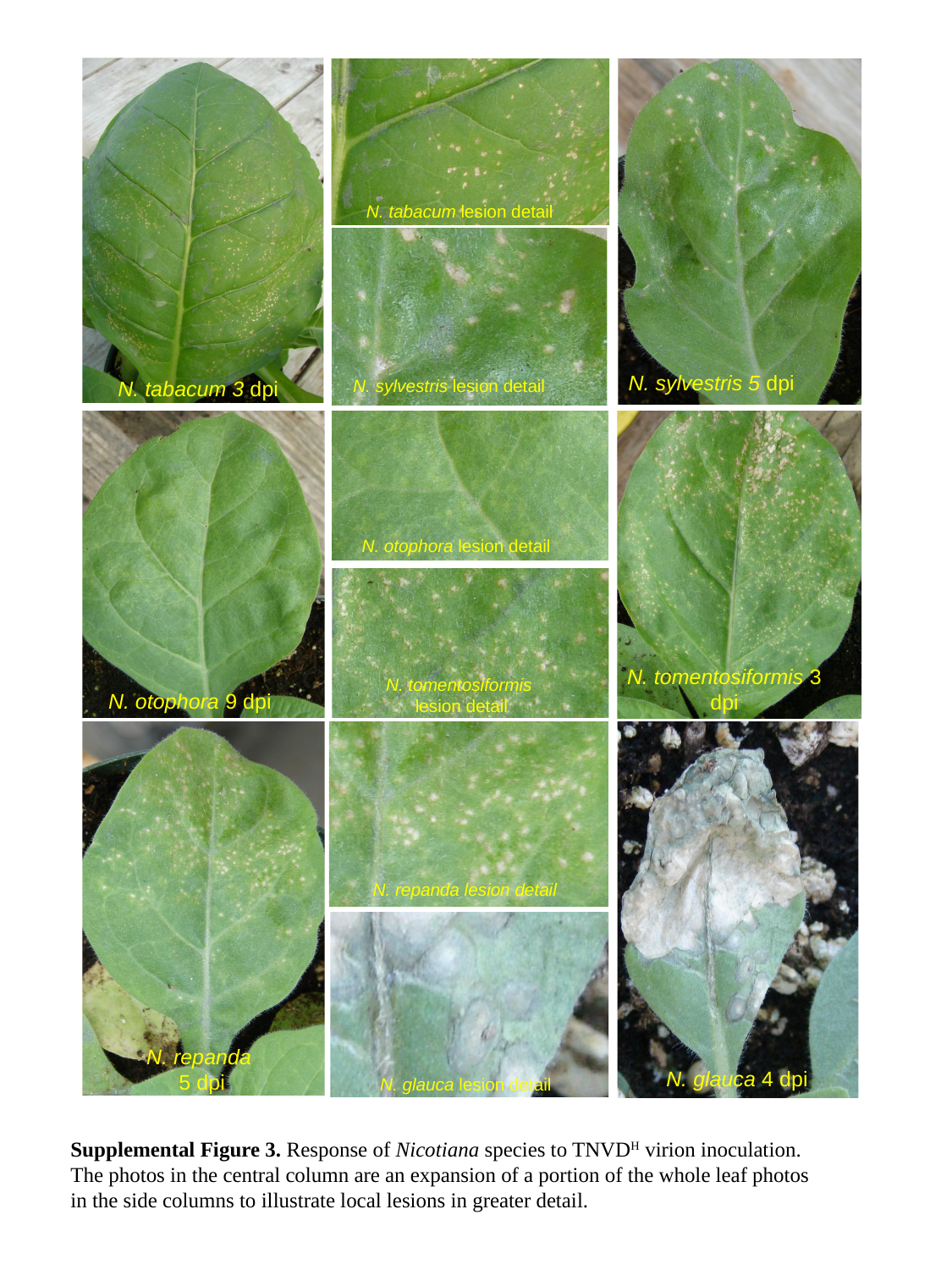

N. tabacum lesion detail
N. sylvestris 5 dpi
N. sylvestris lesion detail
N. tabacum 3 dpi
N. otophora lesion detail
N. tomentosiformis 3 dpi
N. tomentosiformis lesion detail
N. otophora 9 dpi
N. alata lesion detail
N. repanda lesion detail
N. repanda
5 dpi
N. glauca 4 dpi
N. glauca lesion detail
Supplemental Figure 3. Response of Nicotiana species to TNVDH virion inoculation. The photos in the central column are an expansion of a portion of the whole leaf photos in the side columns to illustrate local lesions in greater detail.

## Slide 4
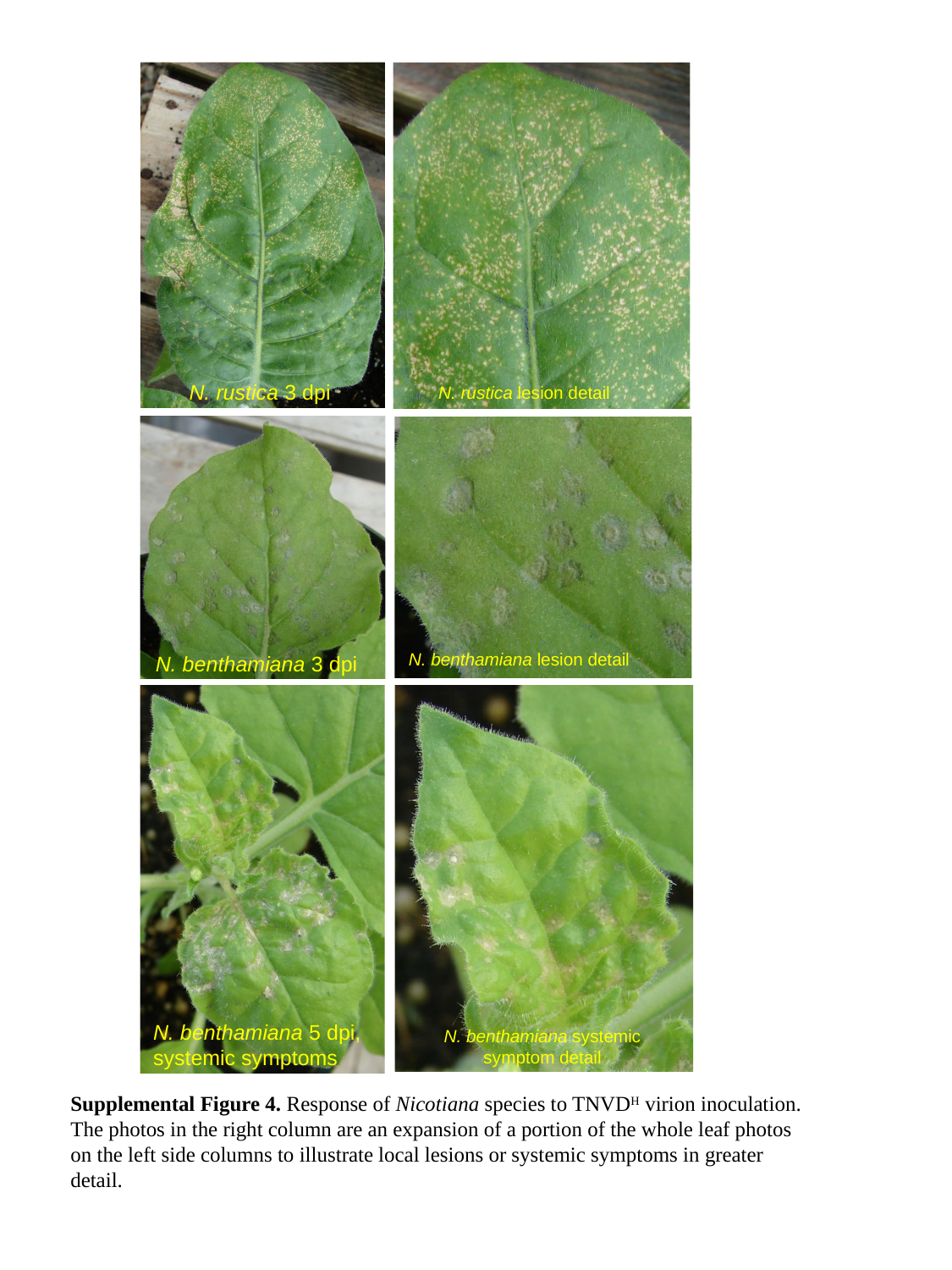

N. rustica 3 dpi
N. rustica lesion detail
N. benthamiana lesion detail
N. benthamiana 3 dpi
N. benthamiana 5 dpi,
systemic symptoms
N. benthamiana systemic symptom detail
Supplemental Figure 4. Response of Nicotiana species to TNVDH virion inoculation. The photos in the right column are an expansion of a portion of the whole leaf photos on the left side columns to illustrate local lesions or systemic symptoms in greater detail.

## Slide 5
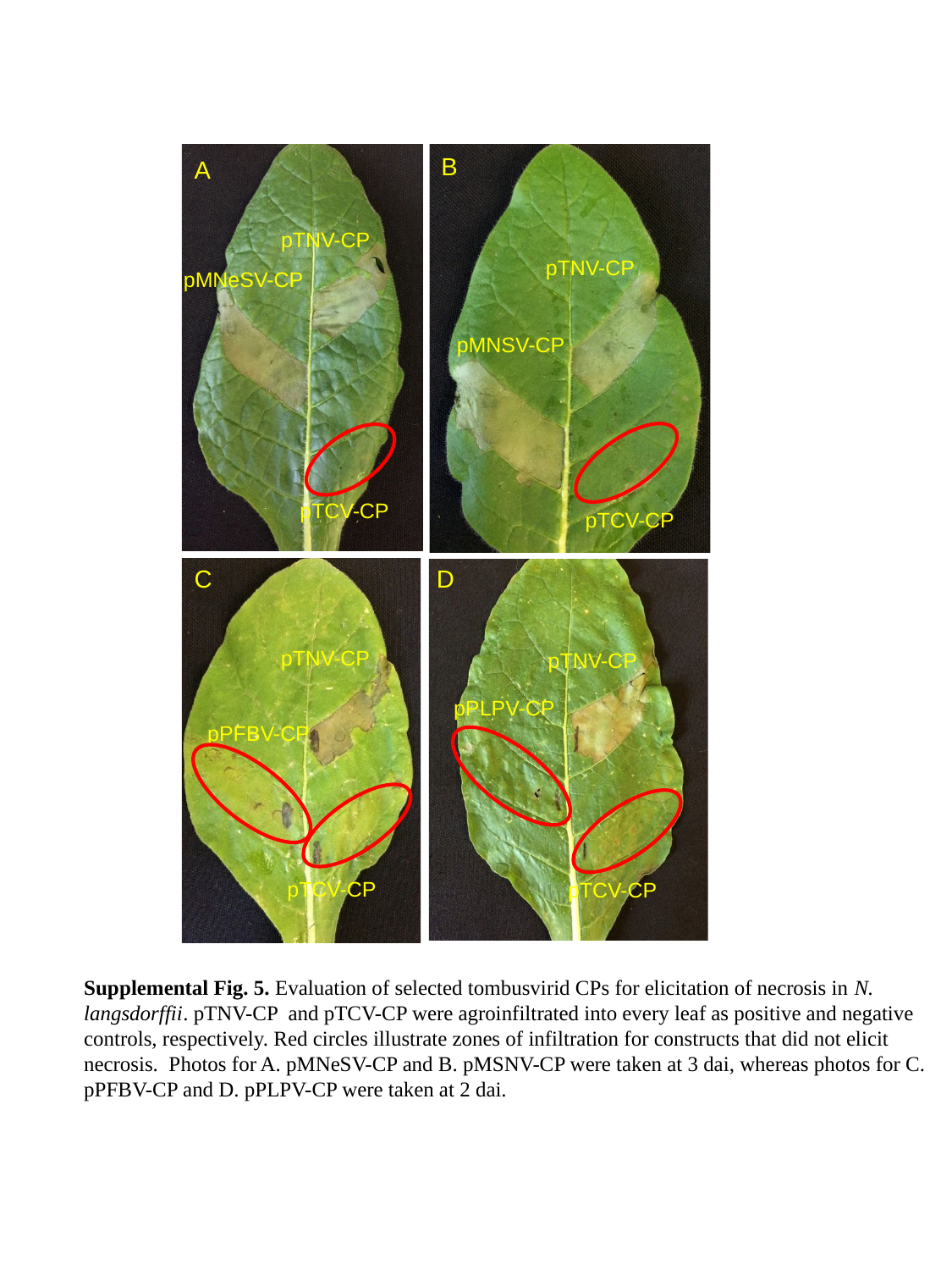

B
A
pTNV-CP
pTNV-CP
pMNeSV-CP
pMNSV-CP
pTCV-CP
pTCV-CP
C
D
pTNV-CP
pTNV-CP
pPLPV-CP
pPFBV-CP
pTCV-CP
pTCV-CP
Supplemental Fig. 5. Evaluation of selected tombusvirid CPs for elicitation of necrosis in N. langsdorffii. pTNV-CP and pTCV-CP were agroinfiltrated into every leaf as positive and negative controls, respectively. Red circles illustrate zones of infiltration for constructs that did not elicit necrosis. Photos for A. pMNeSV-CP and B. pMSNV-CP were taken at 3 dai, whereas photos for C. pPFBV-CP and D. pPLPV-CP were taken at 2 dai.

## Slide 6
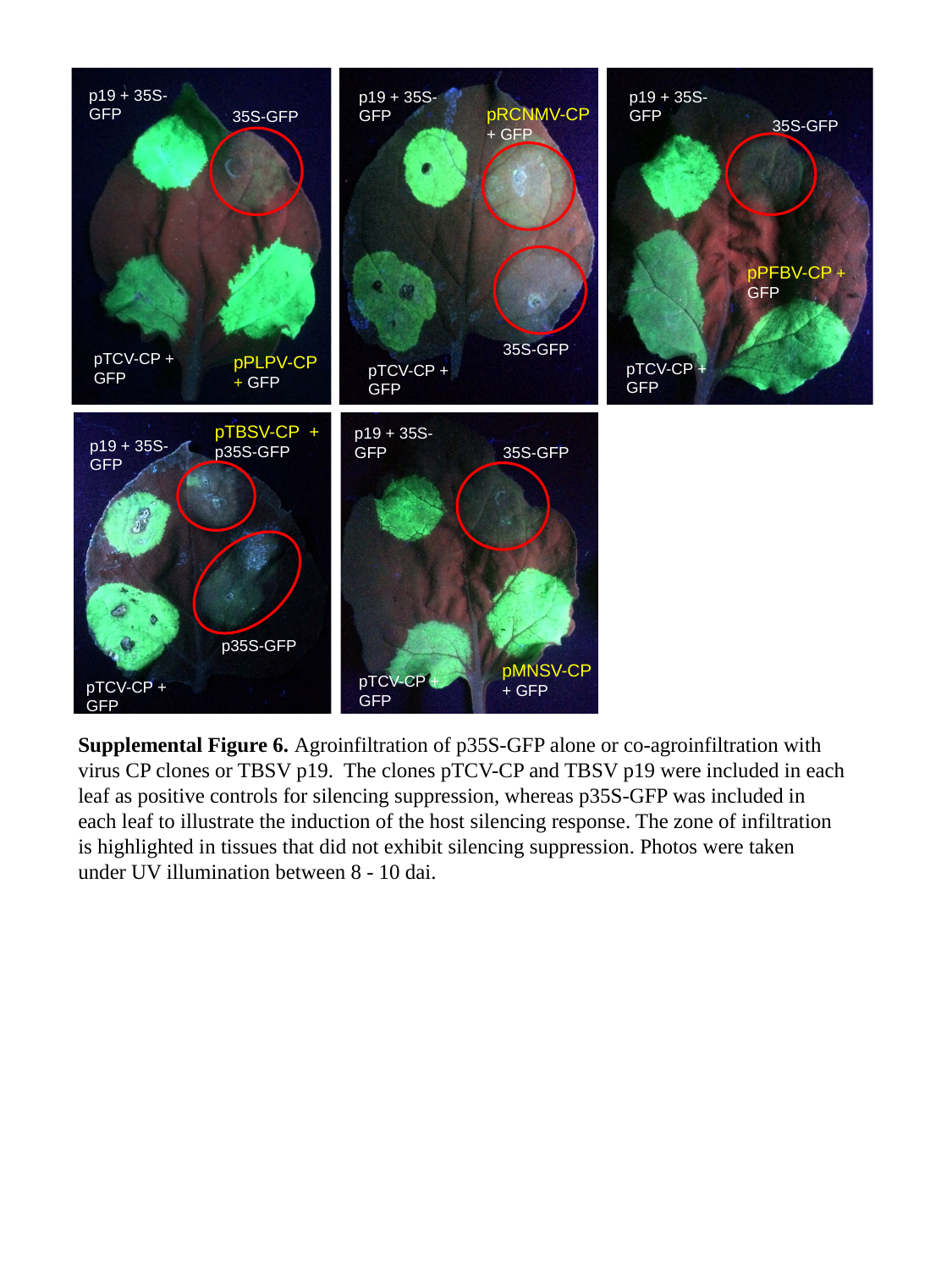

p19 + 35S-GFP
p19 + 35S-GFP
p19 + 35S-GFP
pRCNMV-CP
+ GFP
35S-GFP
35S-GFP
pPFBV-CP +
GFP
35S-GFP
pTCV-CP + GFP
pPLPV-CP + GFP
pTCV-CP + GFP
pTCV-CP + GFP
pTBSV-CP +
p35S-GFP
p19 + 35S-GFP
p19 + 35S-GFP
35S-GFP
p35S-GFP
pMNSV-CP
+ GFP
pTCV-CP + GFP
pTCV-CP + GFP
Supplemental Figure 6. Agroinfiltration of p35S-GFP alone or co-agroinfiltration with virus CP clones or TBSV p19. The clones pTCV-CP and TBSV p19 were included in each leaf as positive controls for silencing suppression, whereas p35S-GFP was included in each leaf to illustrate the induction of the host silencing response. The zone of infiltration is highlighted in tissues that did not exhibit silencing suppression. Photos were taken under UV illumination between 8 - 10 dai.

## Slide 7
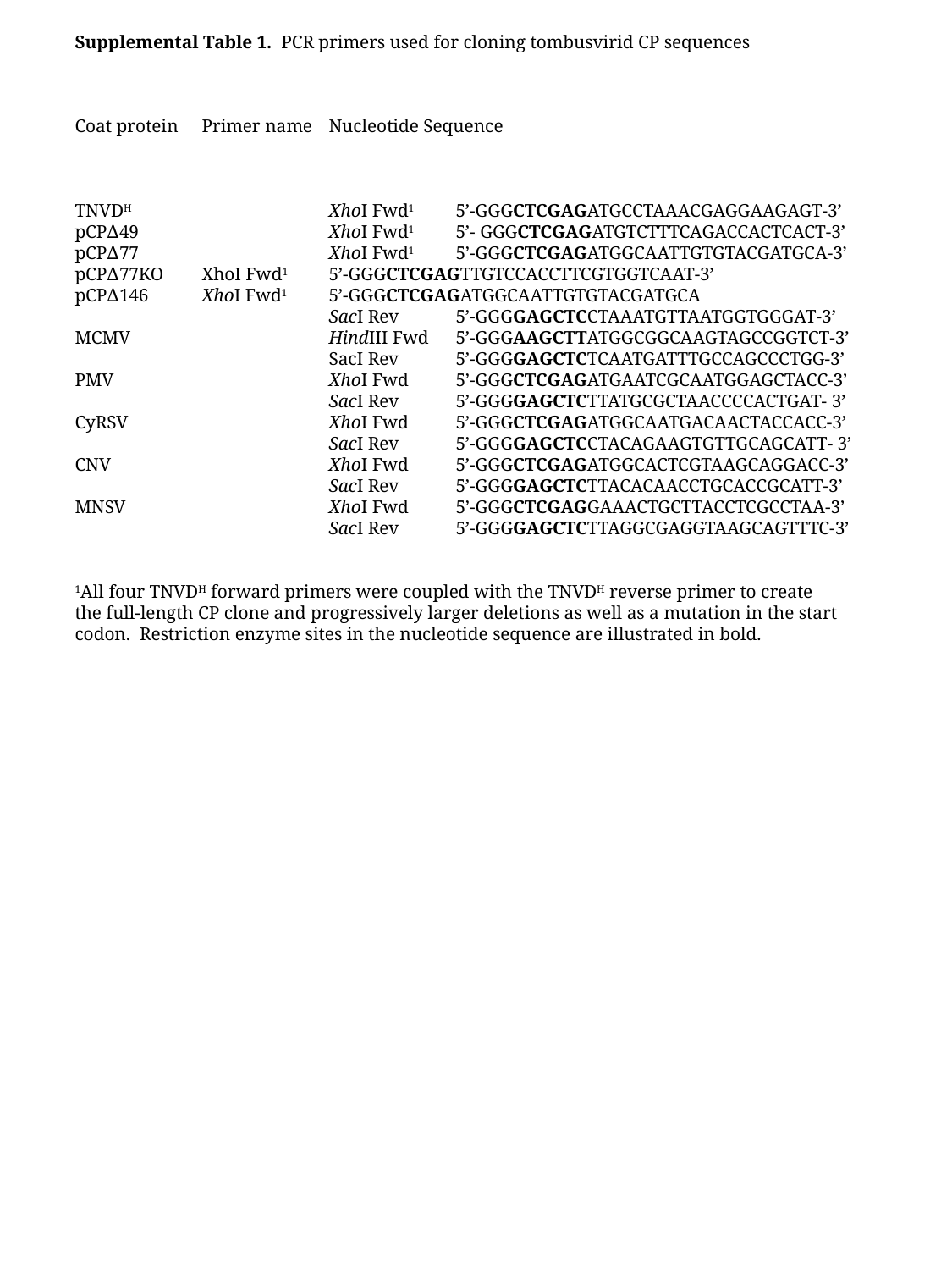

Supplemental Table 1. PCR primers used for cloning tombusvirid CP sequences
Coat protein	Primer name 	Nucleotide Sequence
TNVDH		XhoI Fwd1	5’-GGGCTCGAGATGCCTAAACGAGGAAGAGT-3’
pCP∆49		XhoI Fwd1	5’- GGGCTCGAGATGTCTTTCAGACCACTCACT-3’
pCP∆77		XhoI Fwd1 	5’-GGGCTCGAGATGGCAATTGTGTACGATGCA-3’
pCP∆77KO	XhoI Fwd1	5’-GGGCTCGAGTTGTCCACCTTCGTGGTCAAT-3’
pCP∆146	XhoI Fwd1	5’-GGGCTCGAGATGGCAATTGTGTACGATGCA
		SacI Rev 	5’-GGGGAGCTCCTAAATGTTAATGGTGGGAT-3’
MCMV		HindIII Fwd 	5’-GGGAAGCTTATGGCGGCAAGTAGCCGGTCT-3’
		SacI Rev 	5’-GGGGAGCTCTCAATGATTTGCCAGCCCTGG-3’
PMV		XhoI Fwd	5’-GGGCTCGAGATGAATCGCAATGGAGCTACC-3’
		SacI Rev	5’-GGGGAGCTCTTATGCGCTAACCCCACTGAT- 3’
CyRSV		XhoI Fwd	5’-GGGCTCGAGATGGCAATGACAACTACCACC-3’
		SacI Rev	5’-GGGGAGCTCCTACAGAAGTGTTGCAGCATT- 3’
CNV		XhoI Fwd	5’-GGGCTCGAGATGGCACTCGTAAGCAGGACC-3’
		SacI Rev	5’-GGGGAGCTCTTACACAACCTGCACCGCATT-3’
MNSV		XhoI Fwd	5’-GGGCTCGAGGAAACTGCTTACCTCGCCTAA-3’
		SacI Rev	5’-GGGGAGCTCTTAGGCGAGGTAAGCAGTTTC-3’
1All four TNVDH forward primers were coupled with the TNVDH reverse primer to create
the full-length CP clone and progressively larger deletions as well as a mutation in the start codon. Restriction enzyme sites in the nucleotide sequence are illustrated in bold.

## Slide 8
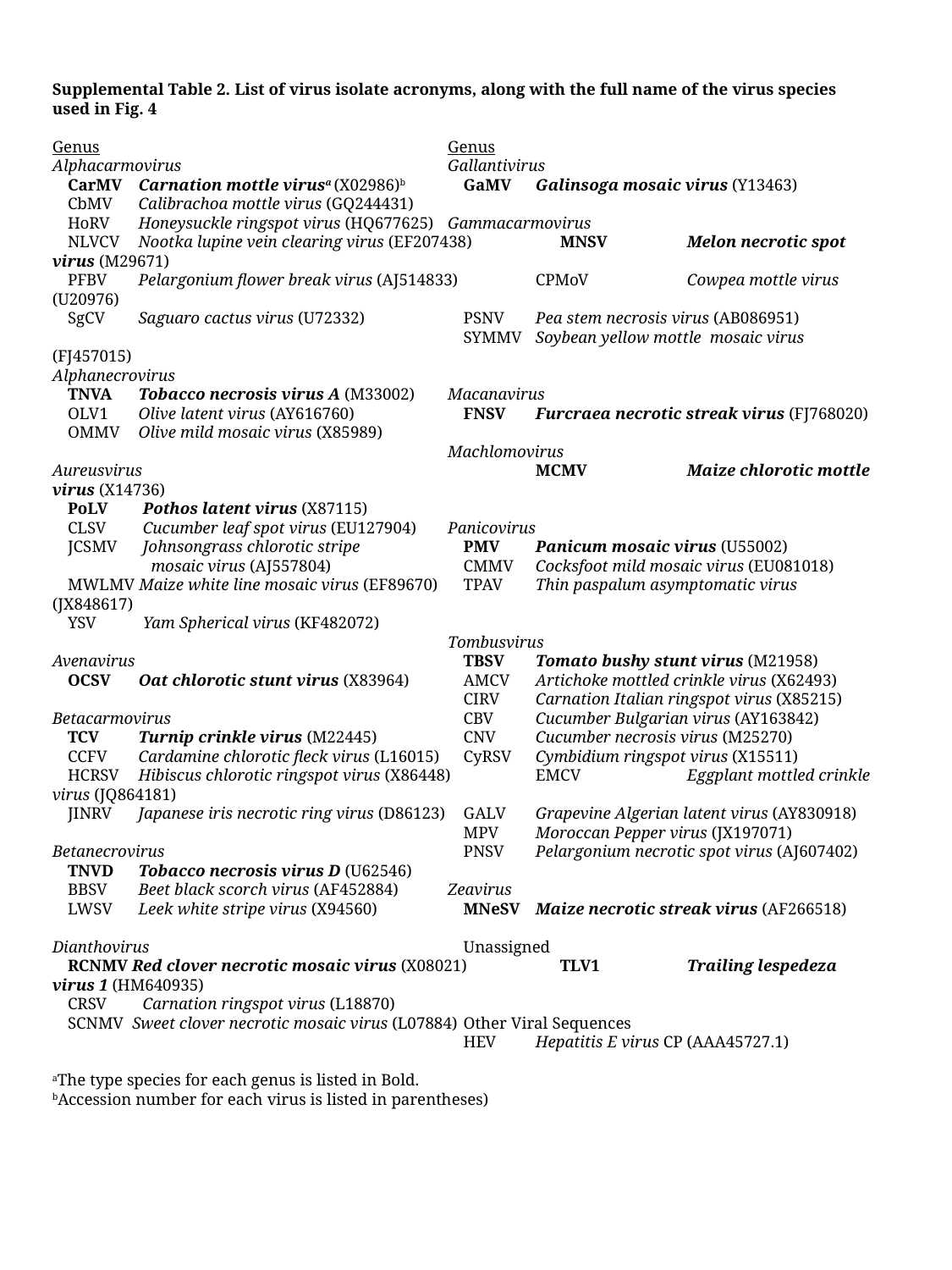

Supplemental Table 2. List of virus isolate acronyms, along with the full name of the virus species used in Fig. 4
Genus		Genus
Alphacarmovirus	Gallantivirus
	CarMV	Carnation mottle virusa (X02986)b		GaMV 	Galinsoga mosaic virus (Y13463)
	CbMV 	Calibrachoa mottle virus (GQ244431)
	HoRV	Honeysuckle ringspot virus (HQ677625)	Gammacarmovirus
	NLVCV 	Nootka lupine vein clearing virus (EF207438)		MNSV	Melon necrotic spot virus (M29671)
	PFBV	Pelargonium flower break virus (AJ514833)		CPMoV	Cowpea mottle virus (U20976)
	SgCV	Saguaro cactus virus (U72332)		PSNV	Pea stem necrosis virus (AB086951)
				SYMMV	Soybean yellow mottle mosaic virus (FJ457015)
Alphanecrovirus
	TNVA	Tobacco necrosis virus A (M33002)	Macanavirus
	OLV1	Olive latent virus (AY616760)		FNSV 	Furcraea necrotic streak virus (FJ768020)
	OMMV	Olive mild mosaic virus (X85989)
			Machlomovirus
Aureusvirus			MCMV	Maize chlorotic mottle virus (X14736)
	PoLV	 Pothos latent virus (X87115)
	CLSV	 Cucumber leaf spot virus (EU127904)	Panicovirus
	JCSMV	 Johnsongrass chlorotic stripe 		PMV	Panicum mosaic virus (U55002)
		 mosaic virus (AJ557804)		CMMV	Cocksfoot mild mosaic virus (EU081018)
	MWLMV Maize white line mosaic virus (EF89670)		TPAV	Thin paspalum asymptomatic virus (JX848617)
	YSV 	 Yam Spherical virus (KF482072)
			Tombusvirus
Avenavirus			TBSV	Tomato bushy stunt virus (M21958)
	OCSV	Oat chlorotic stunt virus (X83964)		AMCV	Artichoke mottled crinkle virus (X62493)
				CIRV	Carnation Italian ringspot virus (X85215)
Betacarmovirus		CBV 	Cucumber Bulgarian virus (AY163842)
	TCV	Turnip crinkle virus (M22445)		CNV 	Cucumber necrosis virus (M25270)
	CCFV	Cardamine chlorotic fleck virus (L16015)		CyRSV	Cymbidium ringspot virus (X15511)
	HCRSV 	Hibiscus chlorotic ringspot virus (X86448)		EMCV	Eggplant mottled crinkle virus (JQ864181)
	JINRV 	Japanese iris necrotic ring virus (D86123)		GALV	Grapevine Algerian latent virus (AY830918)
				MPV 	Moroccan Pepper virus (JX197071)
Betanecrovirus		PNSV	Pelargonium necrotic spot virus (AJ607402)
	TNVD 	Tobacco necrosis virus D (U62546)
	BBSV	Beet black scorch virus (AF452884)	Zeavirus
	LWSV	Leek white stripe virus (X94560)		MNeSV	Maize necrotic streak virus (AF266518)
Dianthovirus		Unassigned
	RCNMV Red clover necrotic mosaic virus (X08021)		TLV1	Trailing lespedeza virus 1 (HM640935)
	CRSV	 Carnation ringspot virus (L18870)
	SCNMV Sweet clover necrotic mosaic virus (L07884)	Other Viral Sequences
				HEV	Hepatitis E virus CP (AAA45727.1)
aThe type species for each genus is listed in Bold.
bAccession number for each virus is listed in parentheses)
